# Attainable and Relevant Moral Exemplars Are More Effective than Extraordinary Exemplars in Promoting Voluntary Service Engagement

**DOI:** 10.3389/fpsyg.2017.00283

**Published:** 2017-03-07

**Authors:** Hyemin Han, Jeongmin Kim, Changwoo Jeong, Geoffrey L. Cohen

**Affiliations:** ^1^Educational Psychology Program, University of AlabamaTuscaloosa, AL, USA; ^2^Department of Ethics EducationSeoul National University, Seoul, South Korea; ^3^Graduate School of Education, Stanford UniversityStanford, CA, USA

**Keywords:** moral exemplar, psychological interventions, moral education, prosocial motivation, attainability, relevance, service engagement

## Abstract

The present study aimed to develop effective moral educational interventions based on social psychology by using stories of moral exemplars. We tested whether motivation to engage in voluntary service as a form of moral behavior was better promoted by attainable and relevant exemplars or by unattainable and irrelevant exemplars. First, experiment 1, conducted in a lab, showed that stories of attainable exemplars more effectively promoted voluntary service activity engagement among undergraduate students compared with stories of unattainable exemplars and non-moral stories. Second, experiment 2, a middle school classroom-level experiment with a quasi-experimental design, demonstrated that peer exemplars, who are perceived to be attainable and relevant to students, better promoted service engagement compared with historic figures in moral education classes.

## Introduction

Stories of moral exemplars have been widely utilized for moral inspiration and moral education. Parents and teachers tell children the stories of historic moral figures, such as Mother Teresa and Martin Luther King Jr., to promote their moral development. In fact, scholars have suggested the positive effects of presenting moral exemplars. According to Kristjánsson's (2006) philosophical account, because a moral exemplar has a better moral character compared to ordinary people, we can recognize our deficiency of a certain moral value by watching exemplars who possess that virtue. This perceived deficiency induces an emotional response, such as envy, that potentially generates motivation to become a better person by emulating the presented moral behavior. Thus, philosophers have endorsed that presenting moral exemplars can promote moral development in moral education (Kristjánsson, 2006; Sanderse, 2013).

We can consider the value of moral exemplars as moral models in moral education from the perspective of moral development. Traditionally, moral development has been considered as the development of moral judgment associated with reasoning skills (Piaget, 1948; Kohlberg, 1981). From this traditional perspective, the development of morality was identified as the sophistication of moral judgment, the formation of a philosophically more sophisticated framework of moral decision-making, i.e., post-conventional framework (Kohlberg, 1981), that can be induced by discussion-based educational methods, such as moral dilemma debates (Blatt and Kohlberg, 1975). However, more recent studies have demonstrated that moral judgment itself does not necessarily induce actual moral behavior (Blasi, 1984); instead, other psychological functions should also cooperate with moral judgment. The Neo-Kohlbergian model introduces a more integrative model of moral functioning to address this issue (Rest et al., 1999). This model includes four functional components: moral sensitivity, moral judgment, moral motivation, and moral character (Rest and Narvaez, 1994). Of these four components, moral motivation deals with whether moral values should be prioritized and implemented in behavior instead of self-oriented values; a person is more likely to behave morally according to her moral decision as she prioritizes moral values over self-oriented values (Aquino and Reed II, 2002; Hardy and Carlo, 2005).

Moral models might play a fundamental role in the development of this aspect of moral functioning, moral motivation. As we mentioned above, from the perspective of traditional moral developmental theory that underscored moral judgment, debates based on hypothetical moral dilemmas would be one of the most recommended moral educational methods (Blatt and Kohlberg, 1975). However, given that hypothetical dilemmas are abstract and detached from everyday human life (Walker and Frimer, 2007; Sommer et al., 2010), they might not effectively promote moral motivation in the real life as sources for moral education. This issue can be addressed by utilizing moral models. Moral models present concrete “real-life” moral goals that are familiar, relevant, and emotionally engaging, and shall be pursued by people (Walker and Frimer, 2007). As a result, the models might be more effective in promoting moral motivation and moral behavior in the real life compared to hypothetical stories. Recent neuroscientific studies of morality support this point. The participants in these studies showed significant neural activity in brain regions associated with moral emotion and moral motivation when they were presented with concrete human beings, not conceptual situations (Greene et al., 2001, 2004; Han et al., 2014, 2016a). Thus, from the perspective of moral development, moral exemplars as moral models can significantly promote moral development, particularly the development of moral motivation and moral behavior.

Social psychological studies also have supported the positive effect of moral exemplars. These effects can be explained by three social psychological mechanisms: vicarious socio-moral learning, moral elevation, and upward social comparison. First, according to social learning theory (Bandura, 1969), moral development in childhood and adolescence is significantly influenced by the presence of moral models. A social psychological experiment conducted by Bandura and McDonald (1963) showed that exposure to moral models indirectly reinforced children's moral behavior and thus, their moral development. Other psychological experiments have replicated this effect of moral modeling (Bandura et al., 1963; Cowan et al., 1969; Prentice, 1972; Dorr and Fey, 1974; Rushton, 1975). Second, recent social psychological studies have suggested that moral elevation provoked by moral exemplars induces moral behavior. Haidt (2000) defined moral elevation as “a warm, uplifting feeling that people experience when they see unexpected acts of human goodness, kindness, and compassion,” which are associated with moral exemplarity. Several social psychological experiments have shown that moral elevation experienced after the presentation of moral exemplars induced various forms of prosocial behavior (e.g., increased motivation for self-improvement Vianello et al., 2010, enhanced fairness and altruistic leadership Algoe and Haidt, 2009). Third, an upward social comparison can also be considered. After people see and compare themselves to moral exemplars, they usually feel negative emotional responses, particularly envy, and are motivated to become morally better people (Wood, 1989; Suls et al., 2002; Schnall et al., 2010). Thus, the stories of moral exemplars perhaps are effective sources for moral education.

However, the presentation of moral stories does not necessarily promote motivation for prosocial behavior and may have detrimental results. Particularly, the presentation of extreme exemplars, such as historical moral figures that do not share any similar skills, experience or background with students, and are difficult to emulate can induce negative emotional and behavioral responses (Monin, 2007). Monin argued that although an upward social comparison to moral exemplars is supposed to induce positive emotional and behavioral responses, people may feel threatened by the comparison when the presented exemplars are perceived to be extreme, such as in the case of moral rebels: those “who take a principled stand against the status quo, who refuse to comply, stay silent, or simply go along when this would require that they compromise their values” (Monin et al., 2008, p. 1). Observers may feel overwhelming inferiority and, as a result, resentment (Monin, 2007). In fact, participants in a social psychological experiment reported negative opinions about moral rebels in the domain of social equity (e.g., racial inequality) and tended to dislike their behavior, such as pursuit of social equity (Monin et al., 2008). A severe discrepancy between the moral behavioral tendency of ordinary people and that of moral exemplars is likely to produce self-conflict, induce self-defense to reduce the conflict, and decrease motivation, as explained by the cognitive dissonance theory (Higgins et al., 1987; Elliot and Devine, 1994; Sherman and Cohen, 2006).

We, thus, consider what types of moral stories can effectively promote prosocial behavior. Given previous psychological studies, two factors, i.e., attainability and relevance, are perhaps associated with the effectiveness. First, attainability can be defined as the perceived possibility of being able to emulate a presented exemplary behavior with a reasonable amount of effort (Lockwood and Kunda, 1997). Previous social psychological experiments have reported that as the degree of perceived attainability increased, participants were more likely to emulate a presented exemplary behavior (Cialdini, 1980; Lockwood and Kunda, 1997). Second, participants were more likely to emulate presented exemplary behavior when the exemplars were perceived to be similar and relevant (Dasgupta, 2011). There are various sources for relevance; for instance, affiliation with the same social or cultural group (Loe et al., 2000; Gino et al., 2009), being in the same age group (Kazdin, 1974; Gould et al., 2003), a shared interest (Lockwood and Kunda, 1997; Lin-Siegler et al., 2016), or even a mere belongingness, such as having the same birthday (Walton et al., 2012), have significantly promoted motivation for emulation.

Unlike the use of extreme moral exemplars, which can backfire, the presentation of attainable and relevant exemplars can effectively promote prosocial behavior. People may think that attainable and relevant exemplars are not significantly different from or extremely better than them (Miller and Prentice, 2012) and they can emulate the exemplars' prosocial and moral behaviors. People might think, “Ordinary people like me have committed to voluntary services and donation behaviors. Why can't I do those things? I can also contribute to our community and world. Even a small contribution helps. It can be appreciated and appropriate,” when they witness attainable and relevant moral stories. In fact, social psychologists who have studied the mechanism of motivation argue that it is needed not only to add motivational force to a tension system but also to lower a psychological barrier hindering a behavior that promotes behavioral change (Leventhal et al., 1965, 1967; Leventhal, 1971; Yeager and Walton, 2011; Bettinger et al., 2012). Attainability and relevance are psychological factors that may lower such a psychological barrier.

### Attainability

Attainability is a factor in the effectiveness of the presentation of exemplars. Although the stories of moral exemplars generally are expected to promote prosocial motivation through mechanisms of vicarious social learning and modeling (Bandura and McDonald, 1963; Bandura, 1969), moral elevation (Haidt, 2000), and positive upward social comparison (Tesser, 1991; Smith, 2000; Suls et al., 2002), unattainable moral stories can induce adverse psychological effects, such as resentment (Monin, 2007; Monin et al., 2008). The self-defense mechanism can explain these negative effects (Alicke, 2000). For instance, when a person reads a story of an extraordinary historic moral figure, she might think the presented exemplar is a completely superior person and she cannot possibly carry out such a moral behavior. In this situation, she attempts to change her attitude and isolate herself from moral duty to protect herself, and she experiences negative feelings, such as a sense of inadequacy or lethargy in moral behavior, associated with self-threatening (Wood, 1989; Alicke, 2000; Monin, 2007). As a result of the isolation, she would not initiate the prosocial and moral behavior that was presented by exemplars.

However, attainable exemplars are likely to promote prosocial motivation. Cialdini's (1980) experiment showed that the amount of donated money was significantly greater when an “even a penny helps” message was presented compared with when the researchers merely solicited donation. This short message made donating behavior seem more attainable and effectively promoted motivation to donate. Moreover, Lockwood and Kunda's (1997) experiment showed that the motivating effect of role models for scholastic activity became greater when participants had enough time before their graduation to catch up with the models. Moral psychologists have also reported that presenting students with ideas about moderately better moral functioning compared to their current developmental stage, which were attainable to reach and emulate, promoted the development of moral functioning (Enright et al., 1983). These studies prove that a person is not threatened by stories about attainable moral behavior. Such behavior seems easy to emulate through a feasible amount of effort, allowing the person to believe that she can accomplish the presented moral behavior and that even a small moral behavior is meaningful and valuable. In addition, the perceived similarity in terms of ability also promotes the linking and likelihood of emulation of presented models (Marx and Ko, 2012); such a similarity makes a person believe that she is close to achieving the presented moral behavior, and she can do the behavior like the presented exemplar. Thus, presenting attainable moral stories can effectively motivate moral behavior, unlike presenting stories of historic moral figures that may backfire.

### Relevance

In addition to attainability, relevance is another factor influencing the motivational effect of exemplars. Irrelevant exemplars, particularly historic figures, cannot effectively promote students' prosocial motivation and make them emulate the presented exemplars. Although students usually say that the heart-touching stories of Mother Teresa and Nelson Mandela are wonderful, they might ask, “So what?” Those figures are not in the same age group or from the same background as students, factors that constitute the foundation of relevance (Loe et al., 2000; Gould et al., 2003; Gino et al., 2009). Thus, these moral stories perhaps seem outside the boundary of the students' everyday lives and have no connection with the students, who may feel that they are not responsible for emulating the presented moral behavior. Consequently, such irrelevant moral exemplars cannot effectively motivate moral behavior and may even backfire, as shown by previous social psychological studies (Monin, 2007; Monin et al., 2008).

Instead, relevant exemplars can produce positive effects. Previous psychological studies have shown that possible sources of relevance that significantly promote motivation include sharing the same interest or goal (Lockwood and Kunda, 1997; Lin-Siegler et al., 2016), social, cultural or historical background (Loe et al., 2000; Gino et al., 2009), being in the same age group (Kazdin, 1974; Gould et al., 2003), or even sensing the presence of a mere belonging, such as having the same birthday (Walton et al., 2012). In general, similarity in general background as well as more specific forms of model-observer similarity significantly improve the likelihood of emulation. For instance, perceived similarity in general background is positively associated with the liking and likelihood of emulation of musical models (Hilmert et al., 2006) and models with skills of interest (Rosekrans, 1967). Moreover, the specific forms of similarity in models also positively contributes to the likelihood of emulation; individuals are more likely to emulate behaviors of same-gender models (Kobasigawa, 1968; Wolf, 1973) or similar-aged models (Hicks, 1965; Kornhaber and Schroeder, 1975; Becker and Glidden, 1979) than opposite-gender, or older or younger models. Why does this factor, relevance, significantly influence the motivational impact of exemplars? First, people are more likely to compare themselves to relevant models than to irrelevant models during an upward social comparison. For instance, attractive others are significantly more likely to be compared and influential when they are of the same gender (Zanna et al., 1975; Brown et al., 1992). Moreover, several classroom experiments have shown that students were more likely to compare themselves to and be motivated by presented models when the models were perceived to be similar and relevant to the students (Blanton et al., 1999; Huguet et al., 2001). Thus, the likelihood of upward social comparison is proportional to the degree of relevance between perceivers' selfhood and the presented exemplars, so relevant exemplars are more likely to be influential than irrelevant exemplars are.

Second, we may consider the effects of group dynamics. People usually are motivated to observe and are influenced by their own group norms or cultural standards that establish their in-group identity (Tajfel, 1982; Abrams and Hogg, 1990; Gino et al., 2009). Recent social psychological experiments focusing on which environmental and group factors significantly influence motivation for both desirable and undesirable behavior confirmed this mechanism. Wenzel (2004) found a positive relationship between the degree of perceived group identity, the presence of group norms requiring desirable behavior, and tax law compliance. Gino et al. (2009) reported that people were more likely to emulate immoral behavior when a model of the immoral behavior was part of their same group. Furthermore, previous meta-analyses demonstrated that the presence in the same field or group of an ethical climate and norm (Loe et al., 2000), and interaction with moral peers (Ford and Richardson, 1994) were positively associated with the development of moral judgment. Thus, people are more strongly influenced by and likely to emulate a model's behavior when the behavior is perceived as permitted by their group norm and the model is affiliated with the same group. Therefore, the relationship between relevance and the motivational impact of exemplars can be explained by the group dynamics of group norm and group affiliation.

### The current study

We conducted psychological intervention experiments to test whether exemplars with attainability and relevance, such as peer exemplars, better promoted motivation for moral and prosocial behavior compared with unattainable and irrelevant exemplars, such as historic figures. In the present study, we used engagement in various service and civic activities as an example of prosocial behavior, since service engagement is closely associated with prosocial motivation in general (van Goethem et al., 2014). First, we performed a lab-level experiment to examine whether stories of attainable moral exemplars were more likely than unattainable and non-moral stories to induce participants' service engagement. Second, we conducted a classroom-level quasi-experiment in middle school moral education classes to scale up the lab-level intervention and to implement the intervention design in an actual educational setting. This quasi-experiment tested whether stories of peer exemplars, which possessed both attainability and relevance for the students, more strongly motivated their service engagement compared with extreme moral stories, such as the stories of historic figures.

## Experiment 1

This experiment tested whether the stories of attainable exemplars were more likely to motivate participants' moral behavior, which was measured by their voluntary service engagement, compared with unattainable moral stories and non-moral stories. It aimed to conduct a preliminary and prototypical study for the development of the exemplar-based intervention method and test its effectiveness thorough a small lab experiment.

### Methods

#### Sample and design

Fifty-nine Korean undergraduate students (20 male, 39 female) participated in this intervention experiment. They were recruited through university information bulletins and social network services, such as Facebook. All participants were enrolled in a national university located in an urban area, Seoul Metropolitan Region in Korea. The average age of participants was 22.17 years (*SD* = 5.20 years) and they had an average of 2.95 years of college education (*SD* = 1.25 years). Participants were randomly assigned into three groups: attainable, unattainable and control groups. Nineteen participants were assigned to the attainable group, 18 to the unattainable group and 22 to the control group. Among the 59 participants who initially completed the pre-test survey forms and participated in the intervention session, 54 participants (20 male, 34 female; 18 attainable condition, 17 unattainable condition and 19 control condition) responded to the post-test survey request. The attrition did not differ significantly among the three conditions, $\chi_{\left(2\right)}^{2}$ = 1.16, *p* = 0.56, *V* = 0.07. This study was exempted from IRB review by the Stanford University IRB because it was identified as “research involving educational tests, surveys, interviews, or observation of public behavior.” All participants their written informed consent.

#### Measures

##### Responses to the interventions

Participants' responses to the interventions were measured with a self-report questionnaire that examined whether interventions were delivered and influenced participants as intended. Participants' responses were measured in three dimensions. First, the degree of induced moral elevation, i.e., the degree of the perceived moral elevation after reading presented stories, was measured by asking this question: “Did you morally elevated by stories?” Second, the perceived moral excellence of each exemplary story, i.e., how strongly was a presented exemplar perceived to be morally better, was quantified by this question: “Did you think that persons presented in stories were morally excellent and better compared to yourself?” These two questions were designed to measure whether participants considered that the presented stories were morally exemplary. Third, the perceived difficulty of emulating voluntary service activity presented in a given exemplary story, which was associated with perceived attainability, was sought by asking this question: “Did you think that it would be difficult to emulate the activities of persons presented in stories?” We did not ask this question of the control group because the presented stories to this group, such as news reports, did not intend to induce emulation among students. An answer to each item was anchored to a five-point Likert scale (1 = strongly disagree or extremely unlikely–5 = strongly agree or extremely likely).

##### Voluntary service activity engagement

We measured each participant's actual voluntary service activity engagement at the pre- and post-test survey using a self-report method. To minimize potential social desirability bias, participants were asked to provide the names of charities or organizations, the amount of time they had participated, and the periods of participation (see Supplementary Materials for the reporting form). Furthermore, we checked if participants were truthful about their participation in order to exclude fake information from analysis. We particularly investigated whether the name of organization reported was the same as that registered in the voluntary service organization database or webpage and whether the period of participation was valid. The degree of engagement was quantified in hours per month. For the pre-test, we requested participants to write down the history of their participation for the month immediately preceding the pre-test survey. For the post-test, participants provided their participation history for the month immediately prior to the post-test survey.

#### Procedures

Before conducting the experiment, five attainable, five unattainable and five neutral stories were prepared as reading material. First, five undergraduate students were recruited, who had been actively engaging in various voluntary service activities for more than 3 months. They were asked to describe in 1,000 words their voluntary service activity experiences and how they have felt when they were participating. Five stories were collected and were used for both the attainable and unattainable conditions, after modifying the amounts of engagement, written in hours per week. We set thresholds determining the attainability of a certain story by calculating results from a preliminary survey that asked 35 undergraduate students the following two questions: “How many hours can you donate to charities every week, without any hesitation? (in hours)” (for the threshold for attainable stories), and “What is the maximum amount of time that you can donate to charities every week? (in hours)” (for the threshold for unattainable stories). The mean response was 4.21 h/week (*SD* = 3.69) for the first question, and 7.57 h/week (*SD* = 4.69) for the second question. The present study calculated the thresholds using the following equations.

$$T_{attn}\;=\;\left\lfloor \bar{X}-z_{99\%/2}\frac{\sigma }{\sqrt{n}}\right\rfloor$$

$$T_{unattn}\;=\left\lceil \bar{X}+z_{99\%/2}\frac{\sigma }{\sqrt{n}}\right\rceil \;$$

The calculated threshold for attainable stories was 3 h per week and that for unattainable stories was 9 h per week. Although all stories were collected from college students, the calculated threshold for unattainability was set to cut off the extreme 1% of the distribution, thus, service engagement exceeding this threshold would probably seem very difficult to emulate for ordinary college students. The number of hours of engagement in each story was modified according to these thresholds. For instance, on the one hand, one example of stories presented to the attainable group was a story about a college student who spent an hour per week to tutor disadvantaged elementary children; on the other hand, one example of stories provided to the unattainable group was a story about a college student tutor who taught disadvantaged children for about 15 h per week. For the control condition, five morally neutral news reports were used, such as a story about a baseball player or new technological developments.

Before the intervention session, participants were asked to complete a pre-test questionnaire measuring their service engagement. Interventions were conducted immediately after the pre-test survey. We provided participants with five stories according to their group assignments and if they were assigned to either the attainable or the unattainable group, we asked them to write solicitation letters for their friends' service participation. Then, the participants completed another questionnaire that measured the responses to the interventions. Six weeks after the intervention session, the participants reported their post-test voluntary service engagement information.

### Results

We analyzed participants' responses to interventions by using ANOVA. Furthermore, we used ANCOVA to compare the changes between the three groups in service engagement. We decided to utilize the ANCOVA method, since it can be used for the comparison of post-test means after controlling for pre-test means when data was collected at two time points (Dugard and Todman, 1995).

#### Responses to the interventions

**Figure 1:**
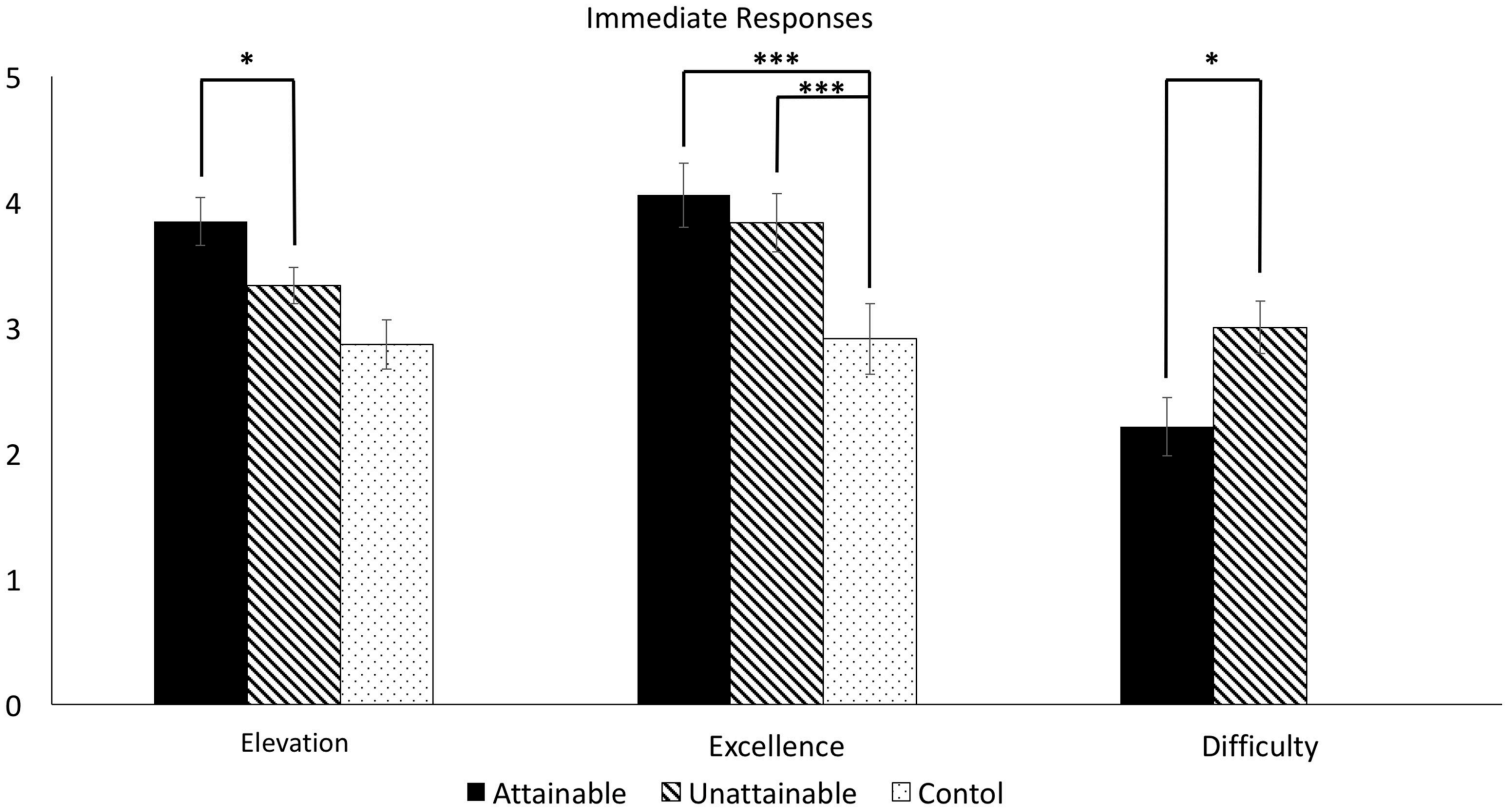
**Participants' responses to interventions in experiment 1**. ^*^p < 0.05, two-tailed. ^***^p < 0.001, two-tailed.

There were significant differences in participants' responses between groups in all three dimensions (see Figure 1). For the induced moral elevation, the main effect of the group factor was statistically significant, *F*_(2, 56)_ = 4.79, *p* < 0.05, η^2^ = 0.11. Only the attainable group reported a significantly stronger response than the control group, *t*_(39)_ = 3.20, *p* < 0.05, 95% CI [0.36, 1.60], *d* = 0.1.01, while there was no significant difference between the unattainable and control groups, *t*_(38)_ = 1.36, *p* = 0.35, 95% CI [−0.23, 1.17], *d* = 0.43. Furthermore, there was no significant difference between the attainable and unattainable groups, *t*_(35)_ = 1.60, *p* = 0.32, 95% CI [−0.13, 1.15], *d* = 0.53. In the domain of perceived moral excellence, the main effect of the group variable was also significant, *F*_(2, 56)_ = 9.89, *p* < 0.001, η^2^ = 0.26. Both attainable *t*_(39)_ = 4.41, 95% CI [0.62, 1.67], *p* < 0.001, *d* = 1.41, and unattainable groups, *t*_(38)_ = 2.97, *p* < 0.01, 95% CI [0.30, 1.55], *d* = 0.97, showed a significantly higher score than the control group, while the difference between attainable and unattainable groups was not significant, *t*_(35)_ = 0.81, *p* = 0.75, 95% CI [−0.33, 0.77], *d* = 0.27. Lastly, the present study compared the degree of perceived difficulty to emulate a presented exemplar between the two moral stories groups. The control group was excluded from this analysis, because the nature of presented stories, such as news reports and sports headlines, was not emulatable. The result of the comparison showed that the attainable group felt their presented stories were significantly easier to emulate than the unattainable group felt about their presented stories, *t*_(35)_ = 2.33, *p* < 0.05, 95% CI [0.10, 1.48], *d* = 0.79.

#### Voluntary service activity engagement

**Figure 2:**
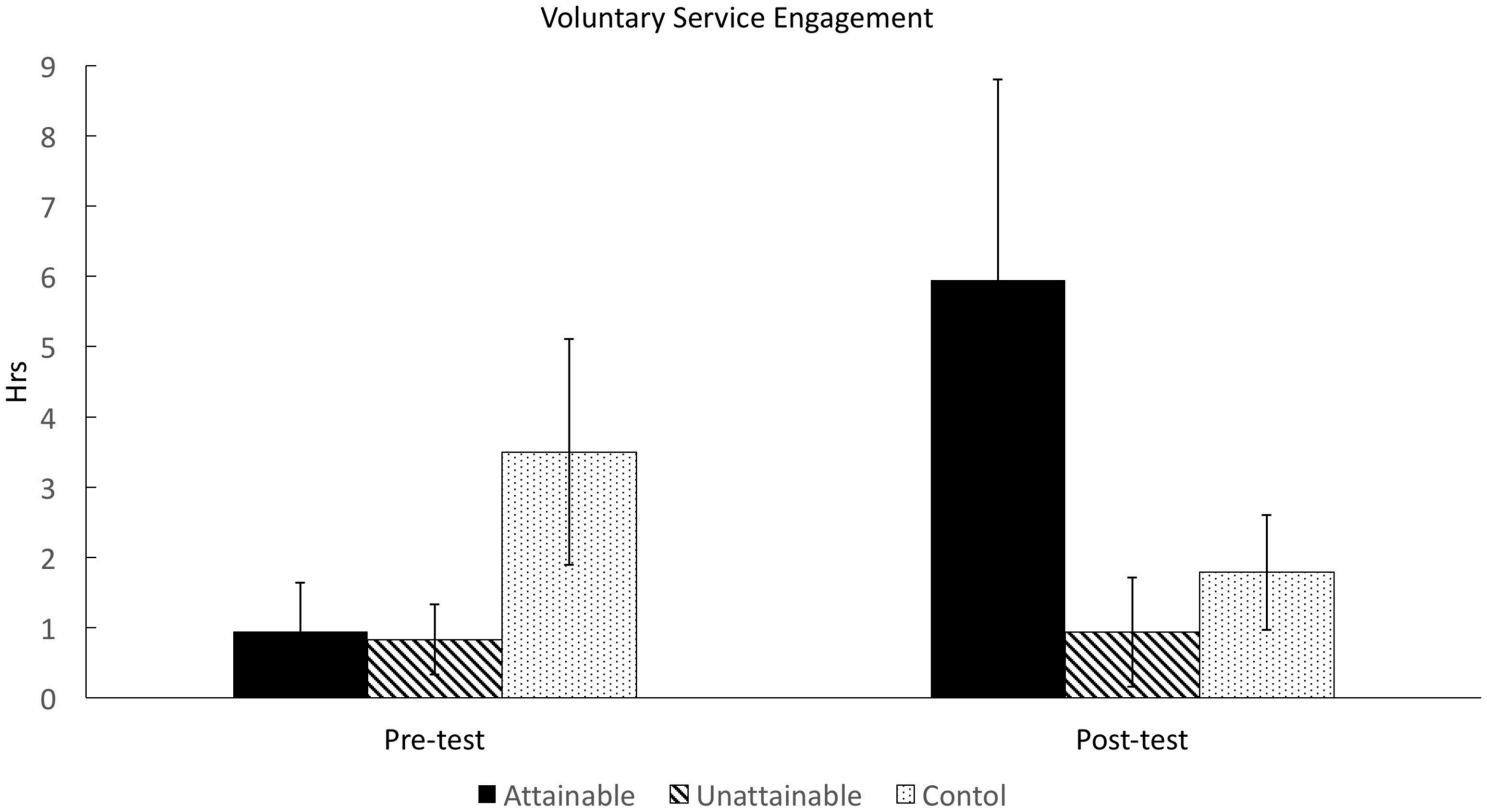
**Participants' voluntary service engagement change (hours per week) in experiment 1**.

First, we conducted preliminary ANOVA to test the random assignment of participants to each group in terms of their pre-test voluntary service engagement. The result indicated that the mean pre-test service engagement did not significantly differ across the three groups, *F*_(2, 56)_ = 1.84, *p* = 0.17, *f* = 0.17. The randomization process was properly performed. Second, ANCOVA and *post-hoc* pairwise comparison using Scheffe's method were used to test whether the attainable stories motivated participants' service engagement significantly better compared with unattainable and non-moral stories. We found a significant main effect of group factor, *F*_(2, 50)_ = 3.19, *p* < 0.05, *f* = 0.27, after controlling for initial engagement (see Figure 2). The results of *post-hoc* tests demonstrated that the longitudinal change in the hours of voluntary service activity engagement was significantly greater in the attainable group than in the control group, *t*_(35)_ = 2.37, *p* < 0.05, 95% CI [0.98, 12.8], *d* = 0.73, while such difference was insignificant between the unattainable and control groups, *t*_(34)_ = 0.96, *p* = 0.73, 95% CI [−2.25, 6.27], *d* = 0.32. However, there was no significant difference between the attainable and unattainable groups, *t*_(33)_ = 1.99, *p* = 0.17, 95% CI [−0.10, 9.87], *d* = 0.65.

**Figure 3:**
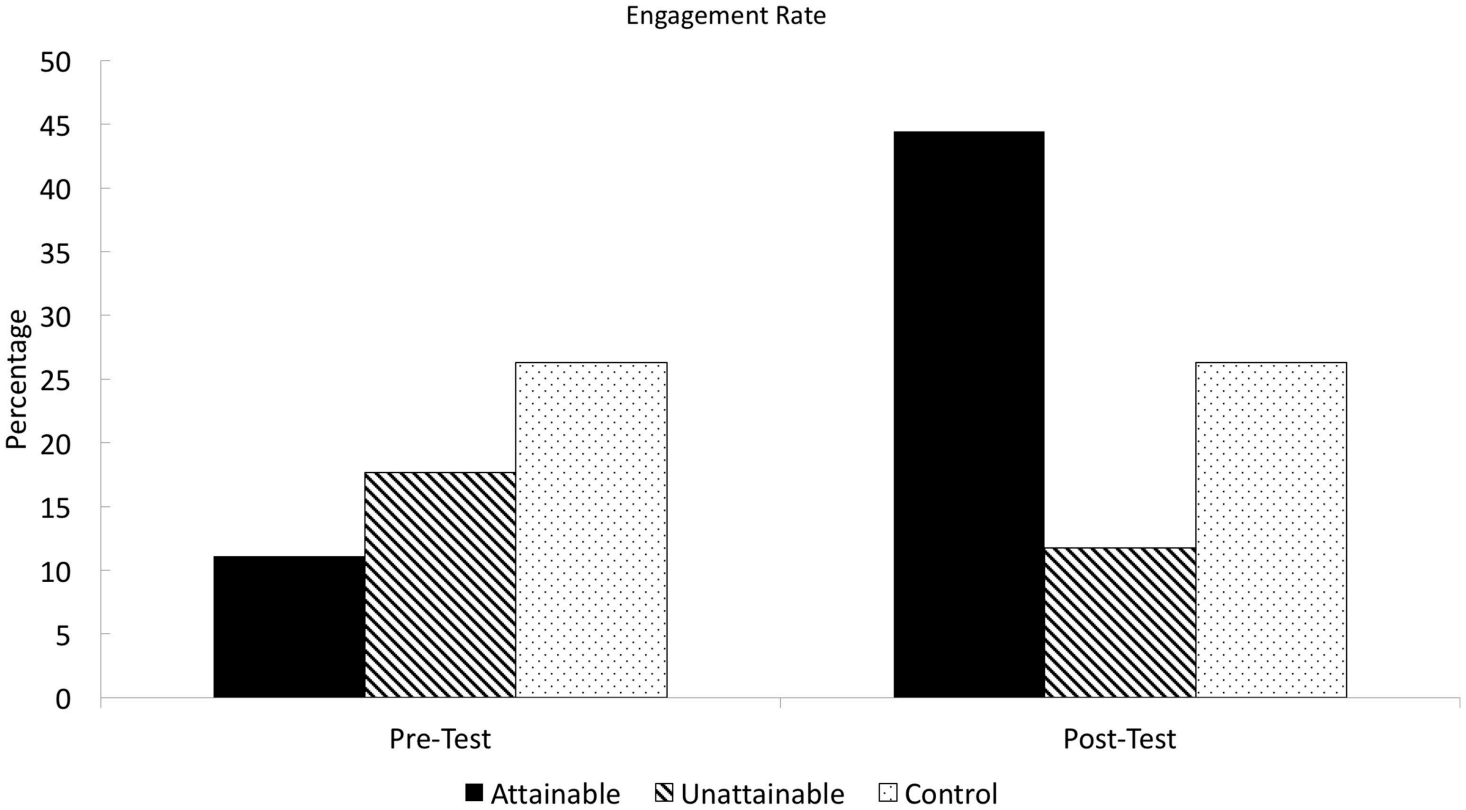
**Participants' voluntary service engagement rate change in experiment 1**.

In addition, the result of logistic regression analysis reported that the post-test engagement, which was coded into a binary variable, was greater in the attainable group than in other groups after controlling for initial engagement (see Figure 3). The difference was statistically significant between attainable and unattainable groups, β = 0.49, *z*_(54)_ = 2.26, *p* < 0.05, 95% CI [0.31, 4.34], and marginal between attainable and control groups, β = 0.31, *z*_(54)_ = 1.68, *p* = 0.09, 95% CI [−0.24, 3.05], given the estimated coefficients. The logistic regression model was also statistically significant, $\chi_{\left(3\right)}^{2}$ = 12.73, *p* < 0.01, *R*^2^ = 0.20, *f* = 0.25.

### Discussion

The results of this experiment supported the hypothesis about the effects of attainable exemplary stories on the promotion of service engagement. The analysis of participants' responses to interventions showed that stories of attainable exemplars induced significantly stronger moral elevation among participants and were more likely to be perceived as morally excellent compared to non-moral stories. Moreover, participants reported that attainable exemplars seemed to be significantly easier to emulate than unattainable exemplars. It suggests that we successfully differentiated the degree of perceived difficulty of emulating presented exemplars between two conditions. The attainable group showed significantly increased engagement compared to the unattainable (only in case of the logistic regression analysis) and control groups. Given these results, attainable stories more effectively promoted participants to initiate and continuously engage in voluntary service activity compared to other types of stories.

However, there are several unanswered questions. First, ANCOVA showed no significant difference in the longitudinal change in service engagement between the attainable and unattainable group. Given the medium effect size (*d* = 0.65), the insignificant difference between those two groups probably derived from the small sample size. In fact, the sample size (*n* = 59) was able to assure the statistical power of 0.80 only when an effect size was large (*d* = 0.80). Thus, an additional experiment with a larger sample size would be required to address this issue. Second, because this experiment was conducted in a lab environment, another classroom-level experiment should be conducted to apply the main idea of the present study to actual moral education.

## Experiment 2

We conducted this quasi-experiment at a middle school with a larger sample to implement the developed interventions in an educational setting. This quasi-experiment tested whether stories of peer moral exemplars, who are close and seem attainable and relevant to students, significantly promoted students' service engagement as a proxy for moral behavioral tendency.

### Methods

#### Sample and design

Given the medium effect size (*f* = 0.27) resulting from the ANCOVA of the previous lab experiment, at least 107 participants were required to assure 80% of statistical power at α = 0.05, which was calculated using the a priori sample size estimation function offered by the G^*^Power package (Faul et al., 2007, 2009). Thus, a total of 111 eighth graders were recruited from four classes at a public middle school located in Seoul, Korea. Of these, 107 students completed both the pre- and post-test surveys. All students were aged 14 years and 50 were females. They were assigned to one of two groups: peer exemplar and historic moral figure groups. Because the students were already assigned to four classes, which had been determined by the school's staff at the beginning of the academic year, the group assignment was not fully randomized. Instead, we randomly selected two classes per condition. As a result, 55 students were assigned to the peer group while 52 were assigned to the historic moral figure group. The attrition did not differ significantly between the two conditions, $\chi_{\_(1)}^{2}$ = 0.38, *p* = 0.54, *V* = 0.06. This study was exempted from IRB review because it was identified as “research involving educational tests, surveys, interviews, or observation of public behavior” by the Stanford University IRB, and “research conducted in established or commonly accepted educational settings involving normal educational practices” by the Seoul National University IRB. All participants their written informed consent.

#### Measures

##### Responses to the interventions

Similar to experiment 1, this quasi-experiment measured students' responses to the interventions after the end of the intervention sessions. We surveyed their responses in three domains identical to experiment 1. They were asked three questions about the degree of moral elevation, perceived moral excellence, and difficulty to emulate. Their answers to each item were anchored to a five-point Likert scale (1 = strongly disagree or extremely unlikely–5 = strongly agree or extremely likely).

##### Voluntary service activity engagement and intention questionnaires

We measured students' service engagement and intention using a questionnaire previously used in civic and community service purpose studies (Bundick et al., 2006; Malin et al., 2015). First, for service engagement, we surveyed the frequency of participation for the last 2 months in service activity offered by: 1. Religion, 2. Charity, 3. Art, and 4. Child-adolescent-student community organizations. Answers were quantified on a one to five scale (“1. None”—“5. More than once per week”). The overall service participation score was calculated by averaging scores in those four domains. In addition, we surveyed intention to participate in service activity by asking whether students would like to engage in service activity, whether they will engage in service activity, and whether they will seek service activity opportunities. Their answers were anchored to a seven-point Likert scale. Moreover, students who participated in voluntary service activities were requested to submit certificates of participation from the organizations to their homeroom teacher by the end of the academic year to prove they actually participated.

#### Procedures

**Figure 4:**
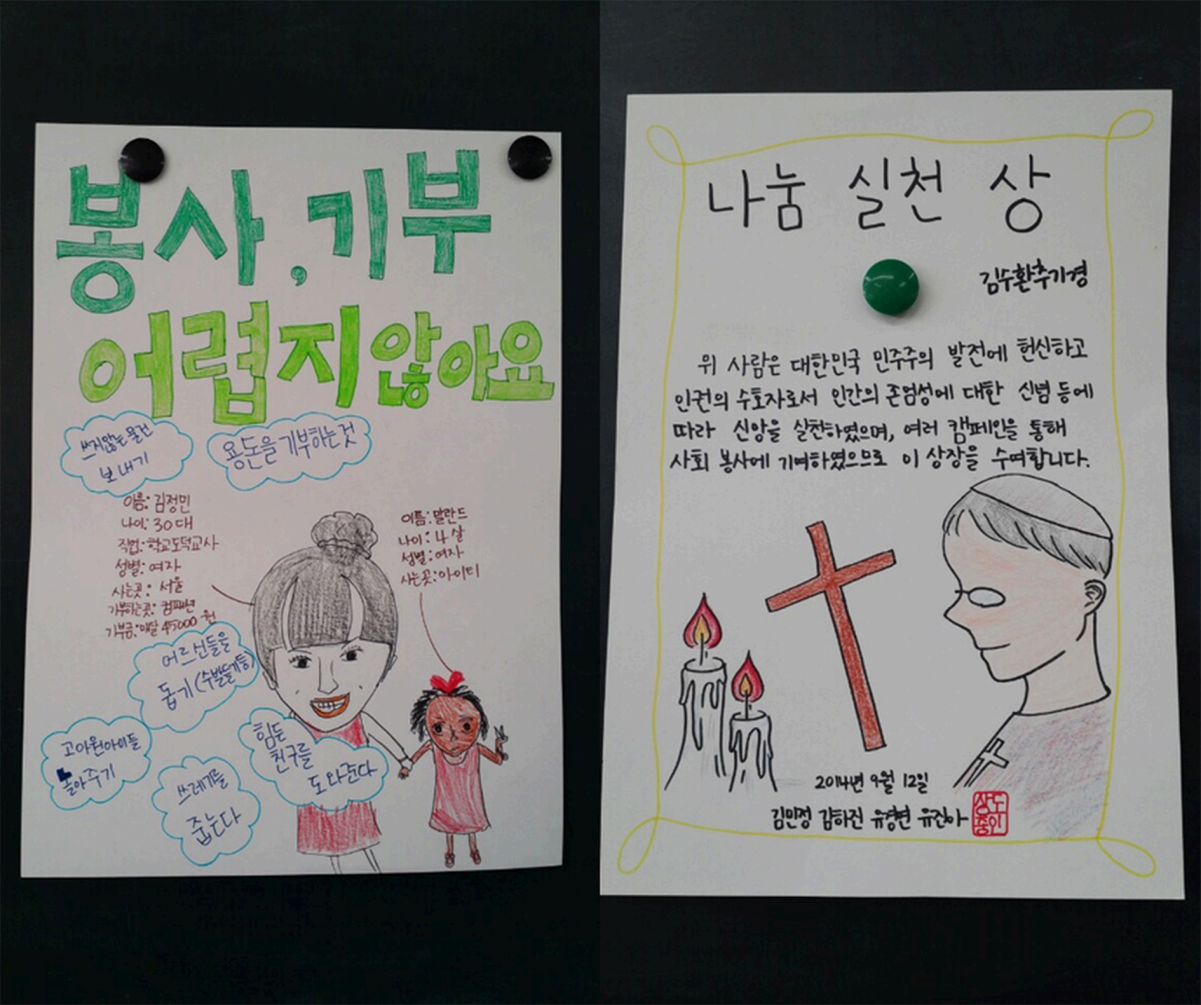
**Students' activity products during moral education classes in experiment 2**. Left: praising voluntary service activity of a teacher exemplar (peer exemplar condition). Right: praising the moral virtue of Stephen Cardinal Kim Sou-hwan (historic moral figure condition).

The educational interventions in this quasi-experiment were conducted as a part of an official subject, Moral Education. This course was required for all eighth graders for 3 h per week. Students completed questionnaires before the initiation of the educational interventions. We initiated the intervention sessions at the beginning of the 2014 fall semester. Students in the peer exemplar group praised and discussed the exemplary behaviors (e.g., donation and voluntary service) of people who were close to them (e.g., family members, friends, teachers, etc.). Students in the historic moral figure group praised and discussed historic and well-known figures, such as Mother Teresa and Martin Luther King Jr. All students participated in various group activities (e.g., poster drawing, award making and focused discussion), and were asked to affirm that they could become morally better people by emulating the exemplars. For instance, on the one hand, a group of students in the peer exemplar group used their teacher who was regularly participating in voluntary service and donating activities as a peer exemplar; on the other hand, a group of students in the historic moral figure group utilized the story of Stephen Cardinal Kim Sou-hwan, who devoted his life to democracy and human right in Korea (see Figure 4). The teacher requested students to autonomously present and share moral stories that they knew with other students. All designed activities were performed in a group setting.

The interventions were conducted once a week (45 min/week) for 8 weeks (see Supplementary Materials for the class plan). All classes were taught by one moral education teacher for consistency in class materials and activities. At the beginning of each session, the teacher, who had 6 years of training in ethics education, gave a 10-min introduction to the virtues that would be discussed during that session. Students autonomously introduced exemplars possessing the designated virtues and engaged for 30 min in various group activities focusing on the exemplars (see Figure 4 for sample products). These student-oriented group activities were designed to assure that all students actually engaged in intervention sessions (see Supplementary Materials for further details pertaining to classroom activities). Twelve weeks after the pre-test survey, students completed the post-test survey measuring their service intention and engagement.

### Results

We performed ANOVA to analyze students' responses to interventions and changes in voluntary service intention and participation.

#### Responses to the interventions

**Figure 5:**
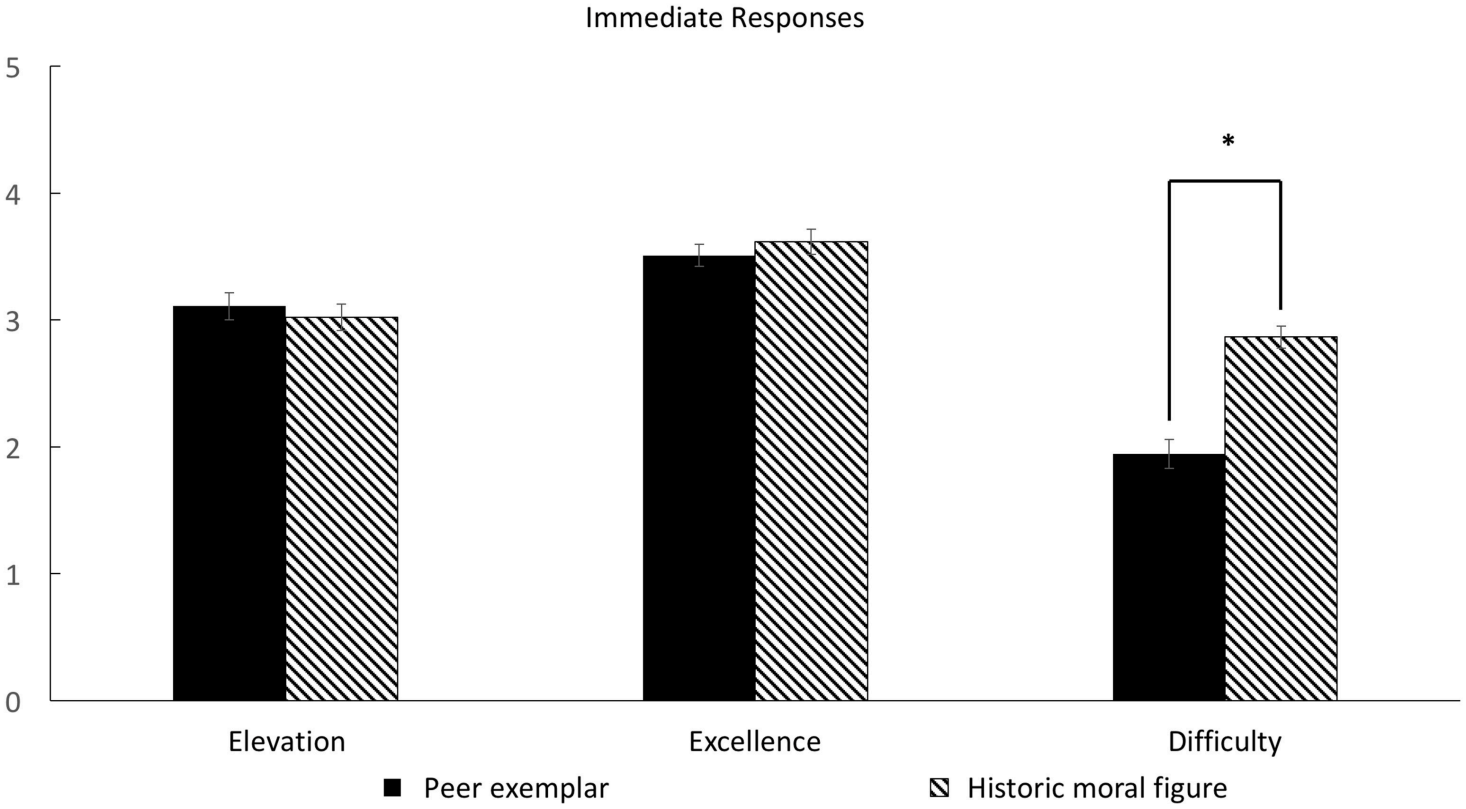
**Students' responses to interventions in experiment 2**. ^*^p < 0.05, two-tailed.

**Figure 6:**
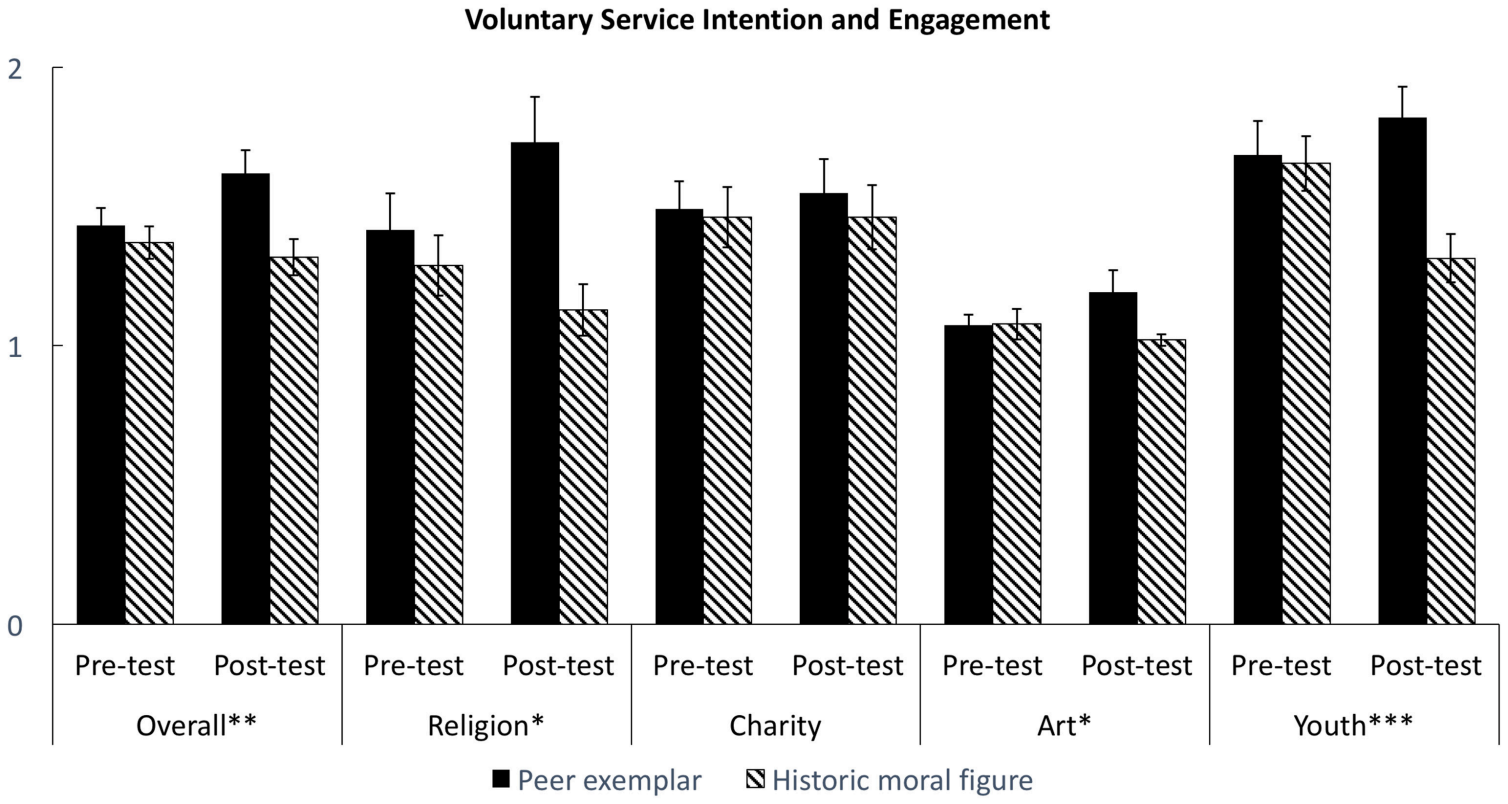
**Students' voluntary service activity engagement change in experiment 2**. ^*^p < 0.05, two-tailed. ^**^p < 0.01, two-tailed. ^***^p < 0.001, two-tailed.

First, there was no significant difference in moral elevation induced by moral exemplars between the two groups, *F*_(1, 105)_ = 0.36, *p* = 0.55, η^2^ = 0.00. Second, the degree of perceived excellence also did not significantly differ between two conditions, *F*_(1, 105)_ = 0.66, *p* = 0.42, η^2^ = 0.01 (See Figure 5). However, peer exemplars seemed significantly easier to emulate compared with historic moral figures, *F*_(1, 105)_ = 38.20, *p* < 0.001, η^2^ = 0.27 (See Figure 6).

#### Voluntary service activity intention and engagement

The pre-test showed no significant intergroup difference in service intention, *F*_(1, 104)_ = 0.11, *p* = 0.74, *f* = 0.00; overall service engagement, *F*_(1, 105)_ = 0.53, *p* = 0.47, *f* = 0.00; engagement in activity offered by religious organizations, *F*_(1, 103)_ = 0.59, *p* = 0.44, *f* = 0.00, charities, *F*_(1, 103)_ = 0.04, *p* = 0.84, *f* = 0.00, art organizations, *F*_(1, 104)_ = 0.00, *p* = 0.96, *f* = 0.00, and youth organizations, *F*_(1, 104)_ = 0.04, *p* = 0.84, *f* = 0.00. This showed the random assignment procedure was appropriately performed.

We conducted a series of ANCOVA to examine the intergroup difference in longitudinal change for each dependent variable. The pre-test score was included in the ANCOVA model to control for baseline differences across individuals. For service intention, the main effect of condition was insignificant, *F*_(1, 103)_ = 1.33, *p* = 0.25, *f* = 0.04. However, we found a significant main effect of the group assignment on overall service engagement, *F*_(1, 104)_ = 8.10, *p* < 0.01, *f* = 0.22 (see Figure 6). The main effect of condition was significant for activity offered by youth community organizations, *F*_(1, 102)_ = 12.30, *p* < 0.001, *f* = 0.31. religion-related organizations the main effect, *F*_(1, 96)_ = 5.65, *p* < 0.05, *f* = 0.17, or art organizations, *F*_(1, 97)_ = 3.94, *p* < 0.05, *f* = 0.16. However, such effect was not significant for activity offered by charity organizations, *F*_(1, 98)_ = 1.00, *p* = 0.51, *f* = 0.32.

Finally, the effect of class factor was insignificant given the results of mixed-effects model analysis. The random effects, i.e., class assignment and student ID, were insignificant, $\chi_{\left(1\right)}^{2}$ = 0.04, *p* = 0.98, while the effect of condition as a fixed effect, *B* = 0.26, *z* = 2.69, *p* < 0.01, 95% CI [0.071 0.45], and the overall model were significant, Wald χ^2^_(2)_ = 52.60, *p* < 001.

### Discussion

Students' responses to interventions showed that although both peer and historic moral exemplars similarly induced moral elevation and were perceived to be morally excellent, the students felt that peer exemplars were significantly easier to emulate than historic moral exemplars. This result was consistent with experiment 1, which showed significant difference between attainable and unattainable groups in the perceived difficulty to emulate presented exemplars. It implies that this quasi-experiment was able to differentiate the perceived difficulty for emulation between two types of exemplary stories. We also showed that close peer moral exemplars presented in moral education classes more effectively promoted service engagement compared with historic moral figures. This is consistent with previous social psychological studies that reported the effect of attainability and closeness on the promotion of motivation (Lockwood and Kunda, 1997; Walton et al., 2012).

Of course, as the historic figures can show a sort of the most developed form of morality, some may argue that such figures might be effective in promoting motivation for emulation by showing moral expertise compared to ordinary exemplars given the previous experiments demonstrating the impact of expertise (Maddux and Rogers, 1980; Klucharev et al., 2008). However, Suls et al.'s (2000) study reported that similar models were more preferred and likely to be emulated by participants compared to expert models, although expert models were more preferred than dissimilar models. Given this, attainability and relevance associated with similarity are powerful sources that promote motivation for emulation, and peer exemplars better promoted service engagement compared to historic figures in our experiment.

Interestingly, the main effect of condition was significant for participation in youth organizations, religion-related organizations, and art-related organizations; the main effect was insignificant in the case of participation in general charities. Since the motivational impact of interventions is maximized when intervention materials include concrete and feasible behavioral options (Leventhal et al., 1965), our interventions would most strongly influence students' engagement in activities that were most prevalent among the peer exemplars. In addition, as middle school students can more easily access youth, religious, or art organizations than general charities focusing on adults, the stories of peer exemplars were more likely to motivate them to engage in youth-related, religious, or art-related organizations, or enable them to maintain their engagement in such organizations at least. However, a significant intergroup difference was not found for voluntary service intention. As previous studies have shown that the development of service intention required a history of actual engagement in service activity (Youniss et al., 2001; Metz et al., 2003), our interventions would not significantly influence service intention during the intervention period. Instead, service intention would change as a result of continued service participation in the long term.

Several unanswered questions should be addressed by future studies. First, it is unclear that which factor produced the significant effect found in this quasi-experiment. Because our class design could not distinguish two factors, i.e., attainability and relevance, the effect of peer exemplars might originate from either or both of them. Although we might be able to argue that “being peer” that is associated with both attainability and relevance generated the motivational effect in general, future studies with a more sophisticated experimental design should be conducted to examine the pure effect of attainability and relevance in educational contexts. Second, this quasi-experiment was not able to randomize fully the class assignment or the group assignment. Because students were assigned to their classes at the beginning of each academic year according to their previous scholastic achievement, behavioral record and socio-economic status (SES), we were able to randomly select only two pre-organized classes per group. Although this group assignment method seems suboptimal, there were alleviating factors. Teachers and school administrators carefully assigned each student to each class to minimize interclass differences in academic performance, behavioral record and SES. Thus, although the class assignment was not completely random, the overall student cognitive and social development and SES would not significantly differ across classes. In addition, because there was no significant pre-test intergroup difference in dependent variables, the interclass difference in service intention and engagement before the intervention period would not be an issue. Second, we were not able to set a pure control group that did not engage in any moral educational activity similar to the control group in experiment 1, since moral education was mandated in Korean middle schools by the national-level standard curriculum (Roh, 2004). All students were required to enroll in moral education classes. Thus, the significant effect found in this quasi-experiment might originate from the mere milieu of general moral educational activity instead of the designed intervention components. Third, we used only one item to ask participants' service engagement, the internal validity of the measurement might be limited. Future studies should utilize more sophisticated measures, such as a reporting form used in Study 1, to address this issue.

## General discussion

We tested the psychological impact of exemplar-applied moral educational interventions in the present study. The stories of attainable and relevant moral exemplars, such as peer exemplars, more effectively promoted students' service engagement compared with those of unattainable and irrelevant moral exemplars, such as historic moral figures.

The utilization of attainable and relevant exemplars can be particularly useful when educators motivate students to initiate prosocial and moral behavior, such as volunteer service activity, at an earlier phase of a moral education class. Because attainable and relevant exemplars promote prosocial motivation mainly by endorsing the value of small moral behaviors (Schein, 1999; Miller and Prentice, 2012), they effectively lower the barrier hindering students' initiation of moral behavior. The concept of scaffolding can also explain the mechanism. This suggests that instead of demanding ideal moral standards and norms immediately, a gradual scaffolding of the concept of morality might be more effective in promoting moral development (Turner and Berkowitz, 2005; Berkowitz, 2011). Likewise, attainable and relevant exemplars that seem to be more easily emulated than historic moral figures can enable students to initiate moral behavior and later to engage in more difficult moral behavior.

Then, should moral educators utilize only attainable and relevant exemplars in classes? Are the stories of Mother Teresa and Nelson Mandela harmful to moral development without any potential benefit? The majority of moral psychologists say “No.” Moral exemplars, as paragons of morality, have been considered valuable sources to investigate morality in real life in developmental psychological studies (Colby and Damon, 1992; Matsuba and Walker, 2004; Walker and Hennig, 2004; Damon and Colby, 2013). Thus, extraordinary moral exemplars, such as historic moral figures, can show students what goals should be pursued in our lives, and what is the ultimate endpoint of moral development (Han, 2015).

Given previous accounts regarding the value of extreme exemplars in moral education, moral educators may have to consider carefully students' developmental level and present exemplars that correspond to the current developmental level. In fact, both moral psychologists and philosophers have underscored that moral educational methods and materials should be based on students' current developmental status and should gradually expose the students to higher developmental levels and standards (Han, 2014, 2015). Thus, extreme exemplars, who usually seem to be distant from students' moral developmental levels and backgrounds, may have to be presented after the students' motivation is initially ignited by attainable and relevant stories. The extreme exemplars can show higher standards of moral behavior to students, make them compare themselves with those higher standards, and finally motivate them to engage in more difficult moral behavior, once the psychological barrier to moral behavior is lowered by attainable and relevant stories.

## Limitations and future directions

Although we demonstrated the importance of attainability and relevance to the design of moral educational interventions using moral exemplars, future studies should address several limitations. First, our measurements relied on self-report. The most fundamental shortcoming of self-report, that is, social desirable bias (Ito and Cacioppo, 2007), has been a particularly significant issue in moral educational studies. Scholars have warned that self-report is not an optimal measurement for students' moral character and behavior because it is susceptible to deception (Kristjánsson, 2013). Although experiment 1 requested participants to provide details of their service activity record to alleviate this issue, experiment 2 relied strongly on simple self-report due to the limited class time. Therefore, future studies should utilize more direct measurements, such as a classroom observation (Woodhead and Paulkner, 2000) or diary method (Conway and Briner, 2002; Pekrun et al., 2002; Boekaerts and Corno, 2005). In addition, possible options might include research methods focusing on the internal psychological processes of prosocial motivation and behavior, such as the functional and structural neuroimaging method (Kristjánsson, 2007; Han, 2014, 2016b; Han et al., 2016a).

Second, although we suggested how to effectively apply ordinary and extreme exemplars in moral education in general, we did not discuss concrete ways to utilize peer and historic moral stories in classes. Thus, future studies should test and compare the long-term effects of diverse course designs in educational settings. First, as the scaffolding approach suggests (Turner and Berkowitz, 2005), a teacher may gradually increase the degree of the extraordinariness of presented moral stories; at the first phase of classes, attainable and relevant moral exemplars are introduced to arouse students' interest in moral behavior, and then gradually less attainable or relevant, but more extraordinary stories are utilized. Second, both ordinary and extraordinary stories may be introduced for each session dealing with a specific moral virtue; attainable and relevant exemplars are introduced to elicit students' interest, and then extraordinary exemplars are presented to demonstrate moral exemplarity. Lastly, only attainable and relevant exemplary stories from peer exemplars may be introduced during all classes without introducing any extraordinary stories; this design is obviously opposite of the mainstream moral educational theory endorsing the value of extraordinary moral figures as moral paragons. In order to test the effectiveness of each model, including the stability of intervention outcomes in the medium to long terms, it is necessary to conduct long-term, large-scale experiments in educational settings. However, due to the limitation of time and resource, such long-term, large-scale experiments would be difficult to conduct (Brown, 1992). In order to address this issue, researchers and educators may consider simulating outcomes of intervention models through computer simulation based on lab-level data; the computer simulation can provide useful information regarding which type of intervention might produce the best outcome in the reality, and how to design long-term, large-experiments to test actual outcomes of interventions while saving time and resource (Han et al., 2016b).

Third, we could not test the pure effect of relevance due to the experimental design. Experiment 1 tested the effect of attainability. However, experiment 2 tested the influence of peer exemplars, who possess both attainability and relevance simultaneously, instead of the pure effect of relevance. Because experiment 2 aimed to test the effect of peer exemplars and consider how to apply these exemplars in actual educational settings, it was necessary to mix relevance and attainability in the experimental design. Thus, future experiments should compare two experimental conditions, relevant and irrelevant exemplar conditions, while controlling for any potential compounding factor, such as attainability.

Fourth, as the experiments were conducted in Korean schools, there might be possible cultural factors that influenced the findings. In fact, previous studies have demonstrated cross-cultural differences in cognitive and moral functioning between Korean and American participants that might be influenced by socio-cultural and educational differences (Norenzayan et al., 2002; Han et al., 2014). Future studies should be conducted in various countries in order to generalize the findings in the present study.

## Concluding remarks

We tested whether attainable and relevant moral exemplars better promoted prosocial and moral behavior, which was measured by participants' voluntary service engagement, compared with unattainable and irrelevant exemplars. Two experiments, one in a lab environment and another in a classroom, successfully demonstrated that attainable and relevant exemplars better promoted moral behavior. The findings show that attainability and relevance are core factors determining the motivational impact of exemplary stories in moral education, particularly among students who are about to initiate their moral engagement. Therefore, moral educators should consider how to utilize attainable and relevant stories and how to design and organize more effective moral education programs. Future psychological experiments with more sophisticated intervention designs should be conducted to replicate the findings of the present study in diverse educational settings.

## Author contributions

HH contributed to all stages of the research project and writing. JK and CJ contributed to the design and implementation of interventions. GC contributed to the research design, interpretation of findings and writing.

### Conflict of interest statement

The authors declare that the research was conducted in the absence of any commercial or financial relationships that could be construed as a potential conflict of interest.
