# Supplementary material for: Attainable and Relevant Moral Exemplars Are More Effective than Extraordinary Exemplars in Promoting Voluntary Service Engagement
Source: Front Psychol. 2017 Mar 7;8:283. doi: 10.3389/fpsyg.2017.00283 (PMC5339280; doi:10.3389/fpsyg.2017.00283)
Supplement: Supplementary file 1 [file Table1.PDF]

Supplementary Materials

Voluntary Service Engagement Reporting Form

Please write your experience of voluntary services during the last month (from . . . to . . .).

Period (If you are not sure of the exact date, please provide the date as you remember)	Name of the charity	Amount of time (hours)
Ex) May. 5. 2013.	Save the Children	3
(The rest has been omitted)		

Class Plan

Week #	Topics	Sample activities
1	Donation and voluntary service	Explore and praise related exemplars
2		Draw a poster soliciting donation and voluntary service engagement
3	Humility and sharing	Explore and praise related exemplars
4		Make an award for exemplars with humility and sharing
5	Kindness and recognition of mistakes	Explore and praise related exemplars
6		Perform a role-playing practicing kindness and recognition of mistakes
7	Responsibility and voluntary help	Explore and praise related exemplars
8		Write news reports about concrete cases of responsibility and voluntary help
